# The effect of massage on patients with chronic fatigue syndrome: A systematic review and meta-analysis

**DOI:** 10.1097/MD.0000000000037973

**Published:** 2024-05-03

**Authors:** Jingnan Li, Feng Piao, Qiaoqiao Zeng, Huixin Yan, Yunpeng Bi, Shaobo Zhang, Bailin Song

**Affiliations:** a Acupuncture and Massage College of Changchun University of Traditional Chinese Medicine, Changchun, China.

**Keywords:** chronic fatigue syndrome, massage, meta-analysis, systematic review

## Abstract

**Background:**

Chronic fatigue syndrome (CFS) is a long-term and complex chronic disease that seriously affects the physical and mental health and quality of life of patients. Massage, as one of the methods in traditional Chinese medicine, can treat both symptoms and root causes and is widely used to treat CFS. The main purpose is to systematically evaluate the impact of massage therapy on the efficacy and safety of CFS patients, providing a reference for clinical practice.

**Methods:**

By searching for literature published in PubMed, Cochrane Library, Web of Science, Embase, Wanfang Database, VIP Database, and China National Knowledge Infrastructure Database until November 2023, randomized controlled trial studies were selected according to the established inclusion and exclusion criteria. The Cochrane system evaluation manual was used to evaluate the quality of the included studies, and RevMan5.4 software was used for meta-analysis.

**Results:**

32 randomized controlled trials were included, with a total of 2594 CFS patients. Meta-analysis showed that the total score of the fatigue scale (FS-14) in the treatment group, MD = −1.59, 95% CI (−1.84, −1.34), *P* < .00001; Physical fatigue score, MD = −1.30, 95% CI (−1.60, −1.00), *P* < .00001; Mental fatigue score, MD = −0.84, 95% CI (−0.99, −0.72), *P* < .0001]; Effective rate [RR = 1.23, 95% CI (1.19,1.28), *P* < .00001]; all indicators were superior to the control group, Only one study reported adverse reactions, including local swelling, skin bruising, and nausea.

**Conclusion:**

Our research findings suggest that massage therapy has a significant therapeutic effect on CFS, avoiding adverse reactions and improving fatigue symptoms. Therefore, massage therapy for chronic fatigue syndrome should be further promoted and applied.

## 1. Introduction

At present, the prevalence of chronic fatigue syndrome in the global general population ranges from 0.1% to 2.2%.^[1]^ Lim et al’s survey.^[2]^ It shows that the incidence rate of encephalomyelitis in the American population ranges from 0.89% to 1.14%. The incidence rate in the UK is 2%. The survey shows that the prevalence of chronic fatigue syndrome in the Chinese population is 12.54%.^[3]^ Chronic fatigue syndrome (CFS) is a comprehensive disease characterized by persistent and unexplained fatigue, often accompanied by various physical and neurological symptoms, such as fatigue, joint pain, fever, swollen lymph nodes, lack of concentration, insomnia, low mood, and memory loss. However, patients often have no abnormalities in their physical examinations.^[4,5]^ Due to the unclear etiology of chronic fatigue syndrome, patients are often misdiagnosed as depression, anxiety, or panic disorder.^[6]^ Most CFS patients choose to take medication to relieve fatigue.^[7]^ At present, the medical treatment for chronic fatigue syndrome includes cognitive-behavioral therapy and graded exercise therapy, which are believed to effectively alleviate symptoms.^[8,9]^ However, there are certain requirements for the personality traits and psychological qualities of patients, and the suitable population is relatively small.^[10]^ In China, massage, as a traditional Chinese medicine therapy, is widely used in the treatment of chronic fatigue syndrome. Guided by the basic theories of traditional Chinese medicine, diagnosis, and treatment are carried out based on the specific syndrome types of the diagnosed and treated objects. Select the corresponding massage technique and operating area, and stimulate the corresponding area or acupoint through the technique to form a massage prescription.^[11]^ It is particularly suitable for treating chronic diseases and has good therapeutic effects.^[12]^ Currently, more and more researchers are engaged in clinical research related to chronic fatigue syndrome.^[13]^ Therefore, the clinical application and reports of this therapy for treating chronic fatigue syndrome continue to increase.^[14]^ The research on the therapeutic effect of massage on patients with chronic fatigue syndrome is gradually increasing, but there is a lack of comprehensive evidence-based medicine to evaluate the effectiveness and safety of treatment. On the basis of existing clinical trial literature, we conducted a meta-analysis on the safety and effectiveness of tuina treatment for chronic fatigue syndrome from indicators such as effective rate, FS-14 score, and adverse events, providing a scientific basis and theoretical reference for clinical tuina treatment of chronic fatigue syndrome. However, there is currently limited research on evidence-based medicine in this field. This study conducted a meta-analysis on the efficacy and safety of massage therapy in the treatment of chronic fatigue syndrome, providing evidence-based medicine evidence for clinical treatment.

## 2. Methods

### 2.1. Study registration

This system review program will strictly follow the system review and meta-analysis program (PRISMA-P) preferred report items for reporting. The system review program has been registered on the PROSPERO website (the registration number is CRD42023481608). If there are any adjustments during the entire study period, we will promptly revise and update the detailed information in the final report.^[15]^

### 2.2. Eligibility criteria

#### 2.2.1. Types of studies

We will include only randomized controlled trials of massage treatment for chronic fatigue syndrome in the meta-analysis. The language will be limited to English and Chinese.

#### 2.2.2. Types of participants

Our team included participants diagnosed with chronic fatigue syndrome who met the diagnostic criteria for chronic fatigue syndrome revised by the Centers for Disease Control and Prevention in 1994_._^[16]^ Participants’ gender, education level, age, race, severity, and source of cases are not restricted.

#### 2.2.3. Types of interventions

Inclusion criteria

① The experimental group received massage therapy, while the control group received other treatment methods.

② The experimental group should be treated with massage combined with other therapies, while the control group should be treated with the same other therapies as the experimental group.

#### 2.2.4. Types of outcomes

The outcomes include the effective rate, Fatigue Scale-14 (FS-14),^[17]^ and adverse events.

### 2.3. Exclusion criteria

The exclusion criteria areas are as follows:

① The control group is the massage therapy

② Non-randomized controlled trial (RCT) literature such as single-arm research, retrospective analysis, reviews, meetings, expert speeches, etc

③ Lack or incompleteness of research data

④ Their search object is literature on animals or tissue cells

⑤ Repeated publications

⑥ Unable to obtain full-text

### 2.4. Search strategy

The comprehensive electronic search of PubMed, Web of Science, Chinese National Knowledge Infrastructure, Wanfang Database, Embase, Cochrane Library, Chinese Science Citation Database, and Technology Periodical Database from their inception to November 2023 will be conducted by 2 independent reviewers. The search terms include “chronic fatigue syndrome” or “myalgia encephalomyelitis” or “fatigue syndrome” or “fatigue” and “tuina” or “massage” or “acupoint massage”; “massotherapy.” The search strategy details for PubMed are presented in Table 1. Similar terms will be translated into Chinese for Chinese databases. The language will be limited to English and Chinese. As shown in the Table 1.

**Table 1:** Search strategy for the PubMed database.

Number	Search items
1	chronic fatigue syndrome (all fields)
2	myalgia encephalomyelitis (all fields)
3	fatigue syndrome (all fields)
4	Fatigue (all fields)
5	#1 OR #2-4
6	tuina (all fields)
7	massage (all fields)
8	acupoint massage (all fields)
9	massotherapy (all fields)
10	#6 OR #7-9
11	Randomized controlled trial (all fields)
12	Controlled clinical trial (all fields)
13	Randomly (all fields)
14	Randomized (all fields)
15	Randomised (all fields)
16	Random allocation (all fields)
17	Trials (all fields)
18	#11 OR #12-16
19	#5 And #10 And #17

### 2.5. Study selection and management

Firstly, the three review authors (J.N.L., Q.Q.Z., and F.P.) will independently determine the title and abstract of the search date. Endnote X9 software will be used for literature management and record search. Secondly, the two review authors (J.N.L. and F.P.) will read the full text of the preliminary selective articles and select appropriate studies based on the inclusion criteria. Finally, the selected articles will be placed together. If there are any differences in terms of inclusion and exclusion, we will have group discussions.

### 2.6. Data extraction and management

We will use the WPS Office to design a standard extraction form that will contain all necessary information from the selected study. The data will be recorded in a spreadsheet, including first author, publication time, region, sample size, gender ratio, average age, treatment group intervention methods, control group intervention methods, and outcome indicators.

### 2.7. Dealing with missing data

Faced with missing data or unclear research, we contacted relevant authors to obtain missing information before making an exclusion decision. If the true information of the data fails to be obtained, we will exclude it from the analysis.

### 2.8. Risk of bias assessment

The risk of bias will be independently evaluated by two reviewers based on the Cochrane Intervention System Review Manual. Random sequence generation, allocation concealment, participant and personnel blindness, result evaluation blindness, incomplete result data, selective result reports, and other deviations will be evaluated as low risk, high risk, or fuzzy risk in each randomized controlled trial. The results will be reviewed repeatedly and further discussions among all investigators will be conducted to resolve differences.^[18]^

### 2.9. Statistical analysis

Statistical analysis was performed using the Review Manager 5.4.1 software (Cochrane Handbook). The results were reported as the standard mean difference with a 95% confidence interval (95% CI) for continuous outcomes and relative risk (RR) with a 95% CI for dichotomous outcomes. Statistical heterogeneity among studies was analyzed using the *I*^2^ test andχ²test. We used a fixed-effects model in the absence of heterogeneity (χ²test *P* value > .05, and the *I*^2^ test had a value < 50%). Otherwise, a random effects model was used. Subgroup and sensitivity analyses were performed to explore the potential heterogeneities of the studies and to assess potential confounding factors. Publication bias was detected using funnel plots.

## 3. Results

### 3.1. Literature selection

**Figure 1.**
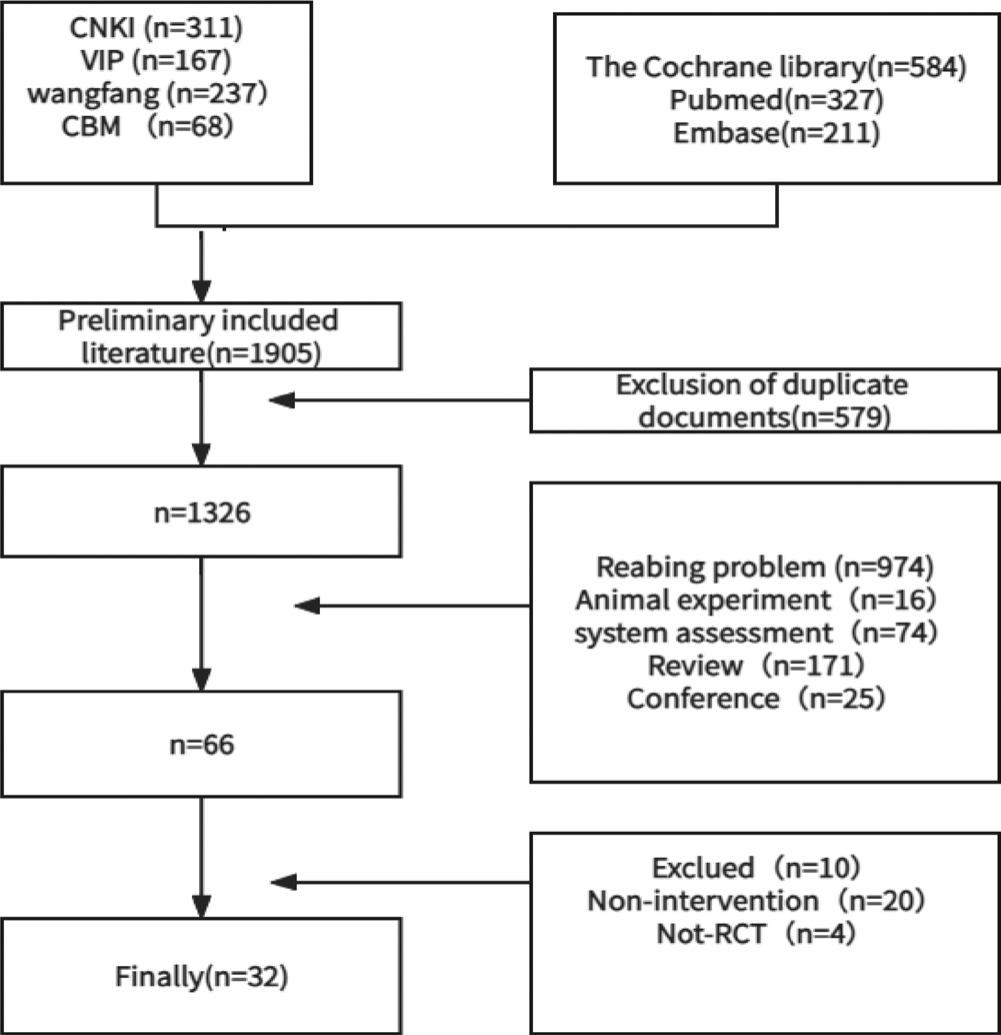
Literature selection.

A total of 1905 articles were retrieved, with 579 duplicate articles excluded. After reading the title, abstract, and full text, systematic reviews, individual cases, animal experiments, and non-RCT articles were excluded. Finally, 32 RCT articles were included. As shown in Figure 1.

### 3.2. Characteristics of included trials

A total of 32 RCTs were included, including,^[19–50]^ The total number of patients with CFS is 2594, Among them, thirteen articles are about massage combined with acupuncture vs acupuncture,^[19,23,24,28,29,31,33–36,38,39,46]^ 2 of them are massages compared to Western medicine,^[42,49]^ 4 articles are about massage compared to acupuncture,^[32,43,45,47]^ 6 articles on massage compared to traditional Chinese medicine,^[21,25,37,40,41,44]^ 7 articles are about massage compared to other therapies,^[20,22,26,27,30,48,50]^ such as moxibustion, hyperbaric oxygen therapy, Nursing treatment, psychotherapy and cupping jar, he basic characteristics of the included study are shown in Table 2.

**Table 2:** Features included in clinical trials.

Author	Time	Area	sample size	Treatment group (male/female)	Control group (male/female)	Disease course (treatment group/control group) (year)	Average age (treatment group/control group) (years)	Treatment group intervention	control group	Outcome indicators
Shen Dongyun^[19]^	2023	China	158	32/47	30/49	(1.467 ± 0.32) (1.465 ± 0.32)	(43.47 ± 7.79) (43.45 ± 7.81)	Massage + acupuncture	acupuncture	①③⑦
Xie Fang^[20]^	2023	China	90	20/25	19/26	-	(40.20.±9.39) (41.00 ± 7.94)	Massage + nurse	nurse	①②
Feng Wei^[21]^	2022	China	40	7/13	8/12	(1.175 ± 0.411) (1.158 ± 0.372)	(38.25 ± 8.69) (36.80 ± 9.75)	Massage	traditional Chinese medicine	①③
Han Zhengqing^[22]^	2022	China	200	80/20	81/19	–	(39.52 ± 2.52) (39.56 ± 2.78)	Massage + cupping jar	cupping jar	①②⑤
Sun Dongwei^[23]^	2022	China	84	17/25	20/22	(1.26 ± 0.27) (1.19 ± 0.26)	(37.12 ± 5.24) (35.67 ± 5.36)	Massage + acupuncture	acupuncture	①②③④⑤
Ren Xiaofei^[24]^	2021	China	86	–	–	–	–	Massage + acupuncture	acupuncture	①④
Shang Kun^[25]^	2019	China	70	17/18	16/19	(2.4 ± 0.15) (2.6 ± 0.13)	(31.00 ± 9.40) (30.00 ± 8.80)	Massage	traditional Chinese medicine	①②⑥⑩
Tang Xianneng^[26]^	2019	China	60	–	–	–	–	Massage + moxibustion	moxibustion	①②③
Feng Qiumei^[27]^	2018	China	26	6/7	7/6		(39.56 ± 2.45) (39.68 ± 2.51)	Massage	hyperbaric oxygen therapy	①
Li Jilei^[28]^	2018	China	57	15/14	14/14	(1.52 ± 0.81) (1.47 ± 0.78)	(33.63 ± 7.05) (33.34 ± 6.98)	Massage + acupuncture	acupuncture	①④
Li Xia^[29]^	2018	China	72	15/21	17/19	(2.50 ± 0.53) (2.40 ± 0.26)	(44.2 ± 10.08) (43.9 ± 10.84)	Massage + acupuncture	acupuncture	①
Wang Jun^[30]^	2018	China	120	32/28	38/22	–	(32.41 ± 6.20) (35.42 ± 5.60)	Massage	psychotherapy	①
Zhang Jie^[31]^	2018	China	68	–	–		–	Massage + acupuncture	acupuncture	①
Wei Guang^[32]^	2017	China	120	29/31	23/27	(1.4 ± 0.80) (1.5 ± 0.60)	(29.05 ± 0.45) (29.08 ± 0.50)	Massage	acupuncture	①②
Yue Caigui^[33]^	2017	China	80	25/15	22/18	–	(31.25 ± 5.41) (32.40 ± 5.46)	Massage + acupuncture	acupuncture	①②
Jiang Haibo^[34]^	2016	China	50	10/15	11/14	–	(32.87 ± 2.57) (33.58 ± 1.34)	Massage + acupuncture	acupuncture	①④
Wang Fei^[35]^	2016	China	60	12/18	14/16	–	(32.49 ± 2.86) (33.16 ± 3.14)	Massage + acupuncture	acupuncture	①④
Fan Yuexia^[36]^	2014	China	86	16/27	18/25	(5.11 ± 3.14) (4.09 ± 4.37)	(44.32 ± 8.15) (45.09 ± 7.53)	Massage + acupuncture	acupuncture	①②
Peng Lei^[37]^	2013	China	120	25/35	24/36	–	(36.0 ± 2.90) (32.0 ± 8.90)	Massage + traditional Chinese medicine	traditional Chinese medicine	①
Xiang Hu^[38]^	2013	China	82	–	–	–	–	Massage + acupuncture	acupuncture	①②
Tian Hengqing^[39]^	2012	China	118	32/36	23/27	(1.59 ± 1.02) (1.65 ± 1.21)	(37.41 ± 5.13) (38.24 ± 4.96)	Massage + acupuncture	acupuncture	①②
Wang Yu^[40]^	2011	China	52	9/17	11/15	(0.941 ± 0.39) (0.915 ± 0.355)	(37.97 ± 10.35) (38.66 ± 11.03)	Massage + traditional Chinese medicine	traditional Chinese medicine	①②
Wu Xingquan^[41]^	2011	China	60	–	–	–	–	Massage	traditional Chinese medicine	①②③
Liu Changzheng^[42]^	2010	China	90	16/14	13/17	(2.26 ± 1.78) (2.21 ± 1.54)	(34.7 ± 4.10) (35.4 ± 3.80)	Massage	Western medicine	①②
Liu Shutian^[43]^	2009	China	68	16/19	16/17	–	–	Massage	acupuncture	①②
Tang Yihong^[44]^	2009	China	100	30/20	33/17	(2.11 ± 0.89) (1.96 ± 0.775)	(37.6 ± 6.7) (36.6 ± 5.5)	Massage	traditional Chinese medicine	①②
Xu Zhao^[45]^	2006	China	60	–	–	–	–	Massage	acupuncture	①
E’ Jianshe^[46]^	2005	China	64	25/8	21/10	–	–	Massage + acupuncture	acupuncture	①
Qi Fengjun^[47]^	2020	China	60	17/13	15/15	(3.14 ± 1.05) (2.40 ± 1.12)	(35.14 ± 3.51) (36.14 ± 4.23)	Massage	acupuncture	③⑧⑨
Xu Yuxin^[48]^	2018	China	73	12/25	10/26	–	–	Massage + psychotherapy	psychotherapy	③⑦
Liang Feng^[49]^	2014	China	40	8/12	9/11	–	–	Massage	Western medicine	③
Yao Fei^[50]^	2012	China	80	–	–	–	–	Massage	psychotherapy	③⑩

① Effective rate ② Recovery rate ③ FS-14 ④ Blood lipid levels ⑤ T cells ⑥ Immunoglobulin ⑦ Pittsburgh sleep quality ⑧ DSI ⑨ SAS 10 FAI adverse reactions SF-36.

### 3.3. Methodological quality of included trials

**Figure 2.**
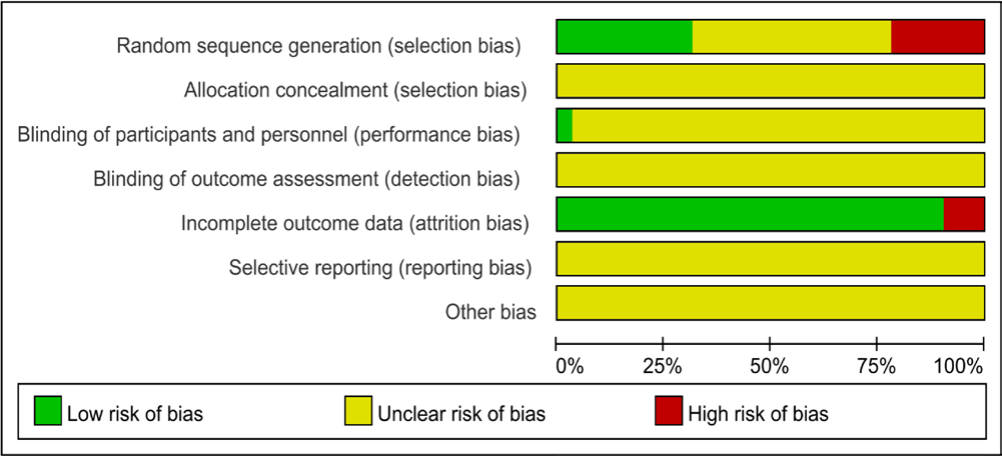
Overall risk of bias.

**Figure 3.**
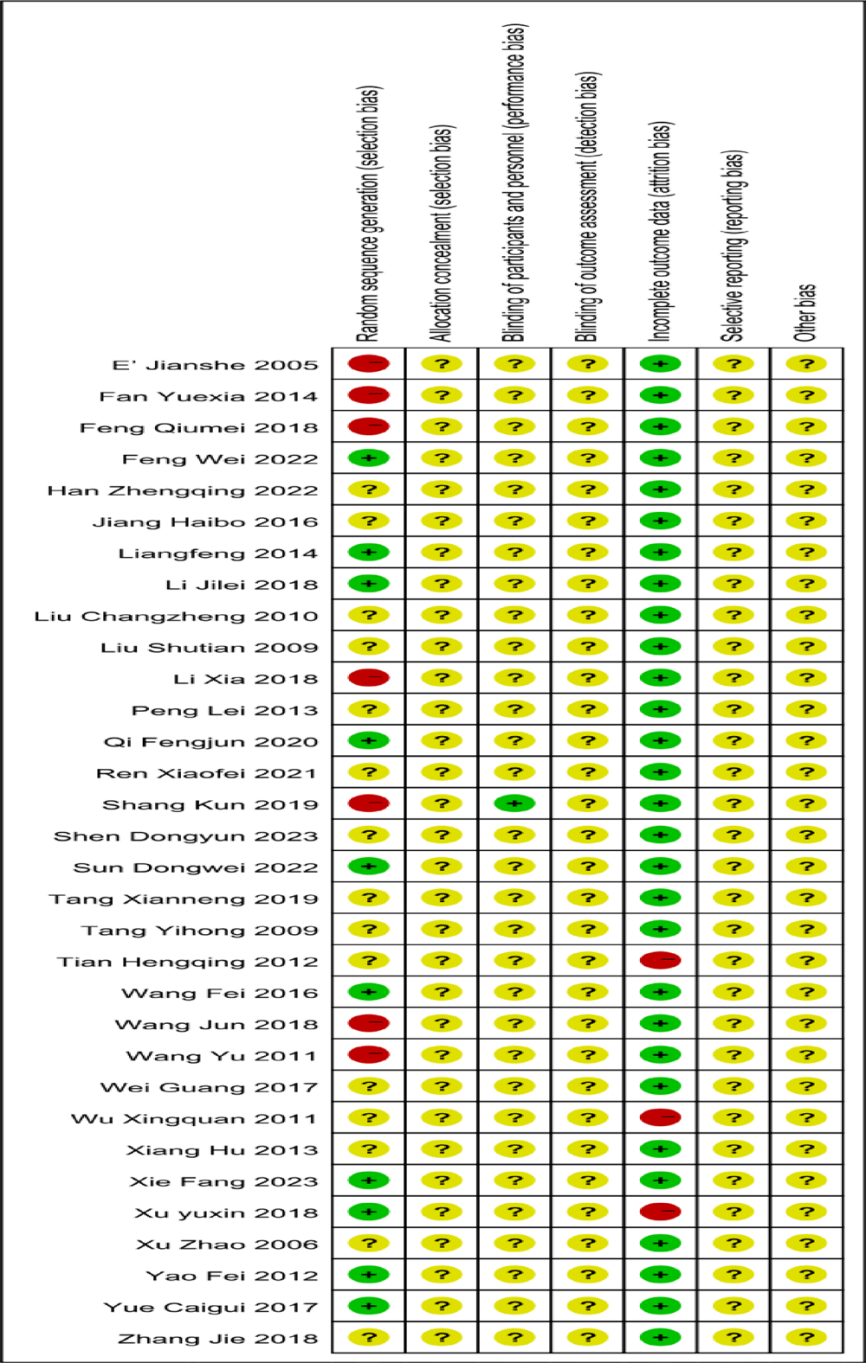
Overall risk of bias.

The generation of quality assessment random sequences included in the study: 10 studies were randomly grouped using a random number table method; Five studies used access order for random allocation, two studies used intervention methods for allocation, one study used drawing lots for random allocation, while the other studies only mentioned the term “randomization.” Allocation concealment: None of the studies mentioned allocation concealment. Blinding by researchers/subjects: Only one study mentioned the use of single blinding for subjects, while the remaining studies did not mention the use of blinding. Selective reporting of research results: No research reports on follow-up status. Integrity of outcome data: Only three studies reported dropout status, and in the remaining included studies, there were no established criteria for termination, exclusion, or dropout, nor were there any reports of dropout. According to the bias risk assessment criteria in the Cochrane Handbook 5.1, evaluate the quality of the included literature, including random sequence generation methods, allocation concealment, blinding, completeness of data results, selective reporting, and other biases. The overall risk of bias in the literature is shown in Figures 2 and 3.

### 3.4. Outcome measures

#### 3.4.1. Effective rate

**Figure 4.**
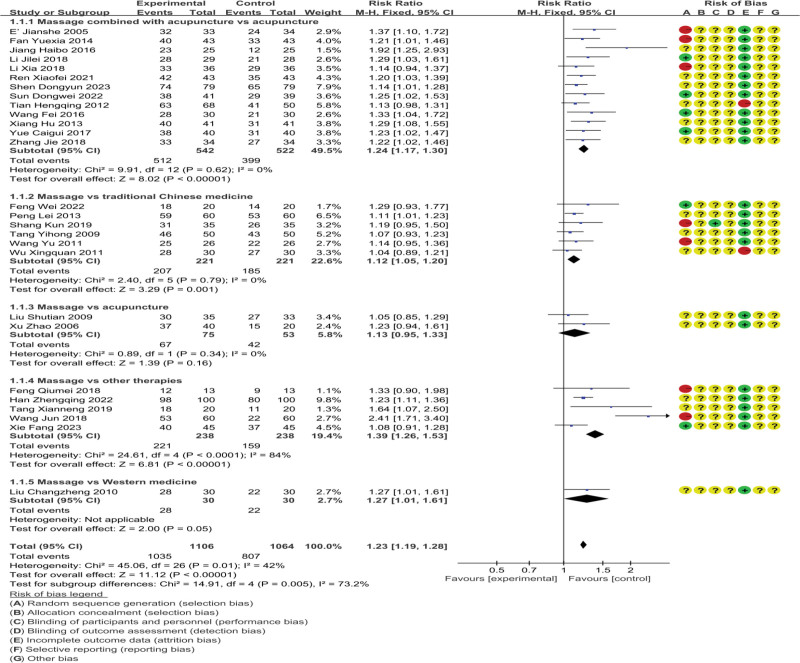
Effective rate.

A total of 28 studies were included, involving 2170 patients.^[19–46]^ The analysis results are shown in Figure 4; The heterogeneity of the included studies is acceptable (*P* = .01, *I*^2^ = 42%), and the statistics are merged using a fixed effects model; The effective rate of massage therapy for chronic fatigue syndrome is significantly better than other therapies(RR = 1.23, 95%CI = 1.19, 1.28), The difference is statistically significant(*P* < .00001). We conducted subgroup analysis based on intervention methods and found that there was a high heterogeneity between subgroups (*P* = .005, *I*^2^ = 73.2%). In the subgroup, massage had a higher heterogeneity compared to other treatment groups (*P* < .00001, *I*^2^ = 84%). We believe this is the source of heterogeneity between subgroups, which is due to the significant differences in intervention methods between the control group and the subgroup, such as hyperbaric oxygen chamber treatment, psychological care, etc., leading to significant differences in efficacy within this subgroup.

#### 3.4.2. FS-14

**Figure 5.**
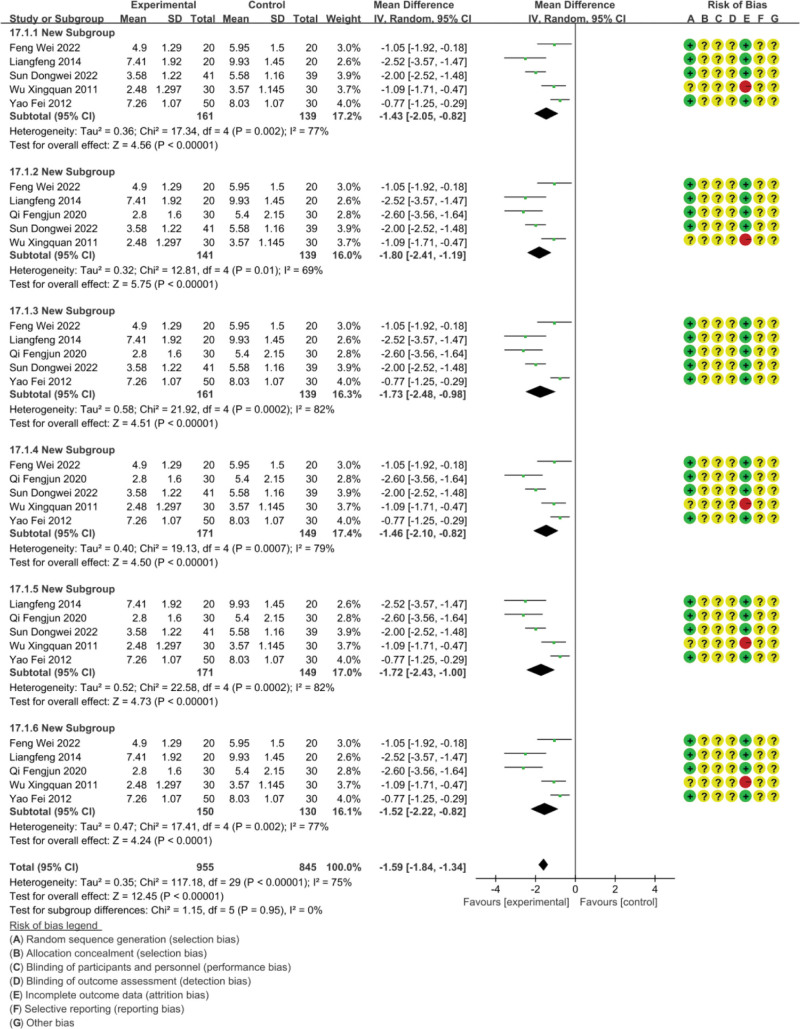
Data graph of FS-14.

**Figure 6.**
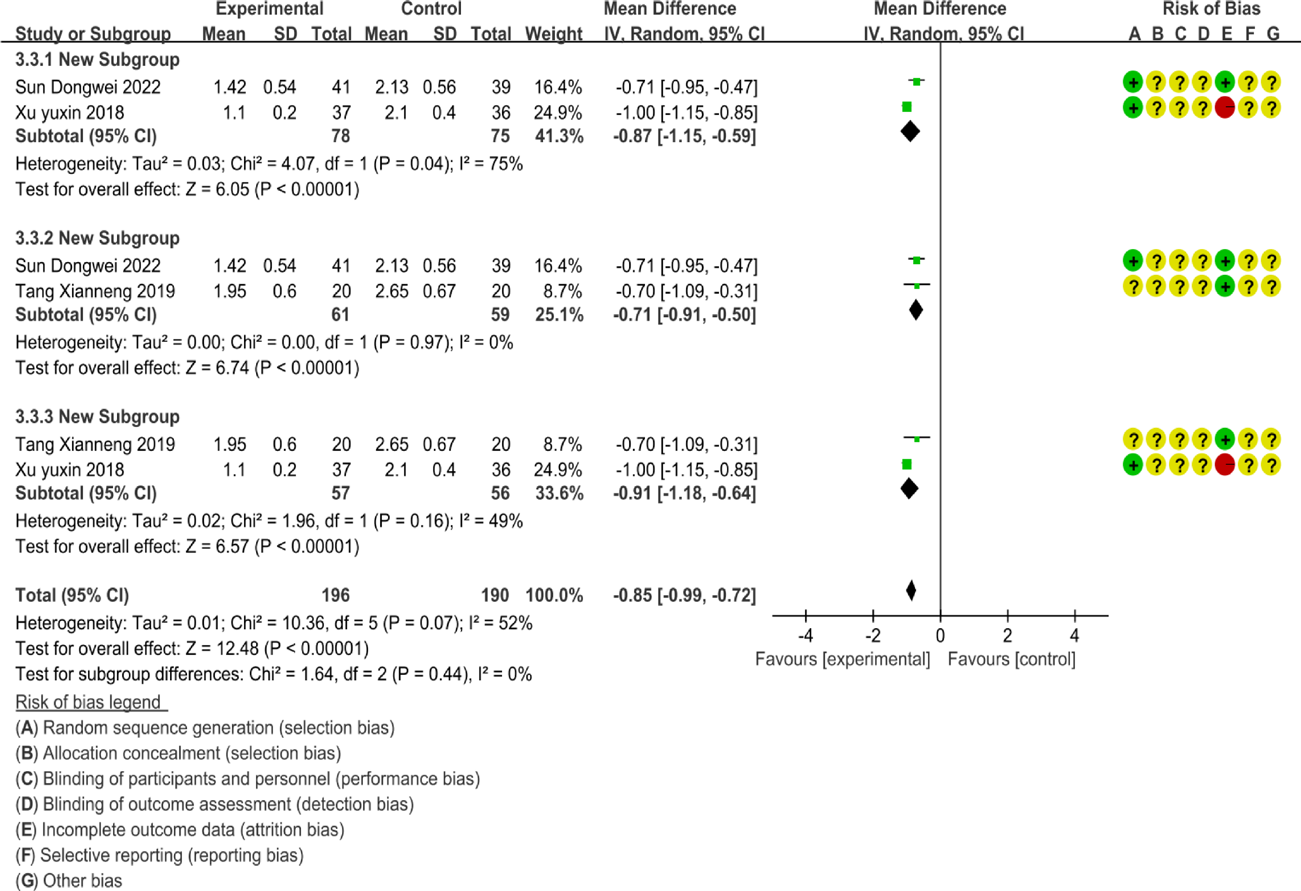
Data graph of FS-14.

**Figure 7.**
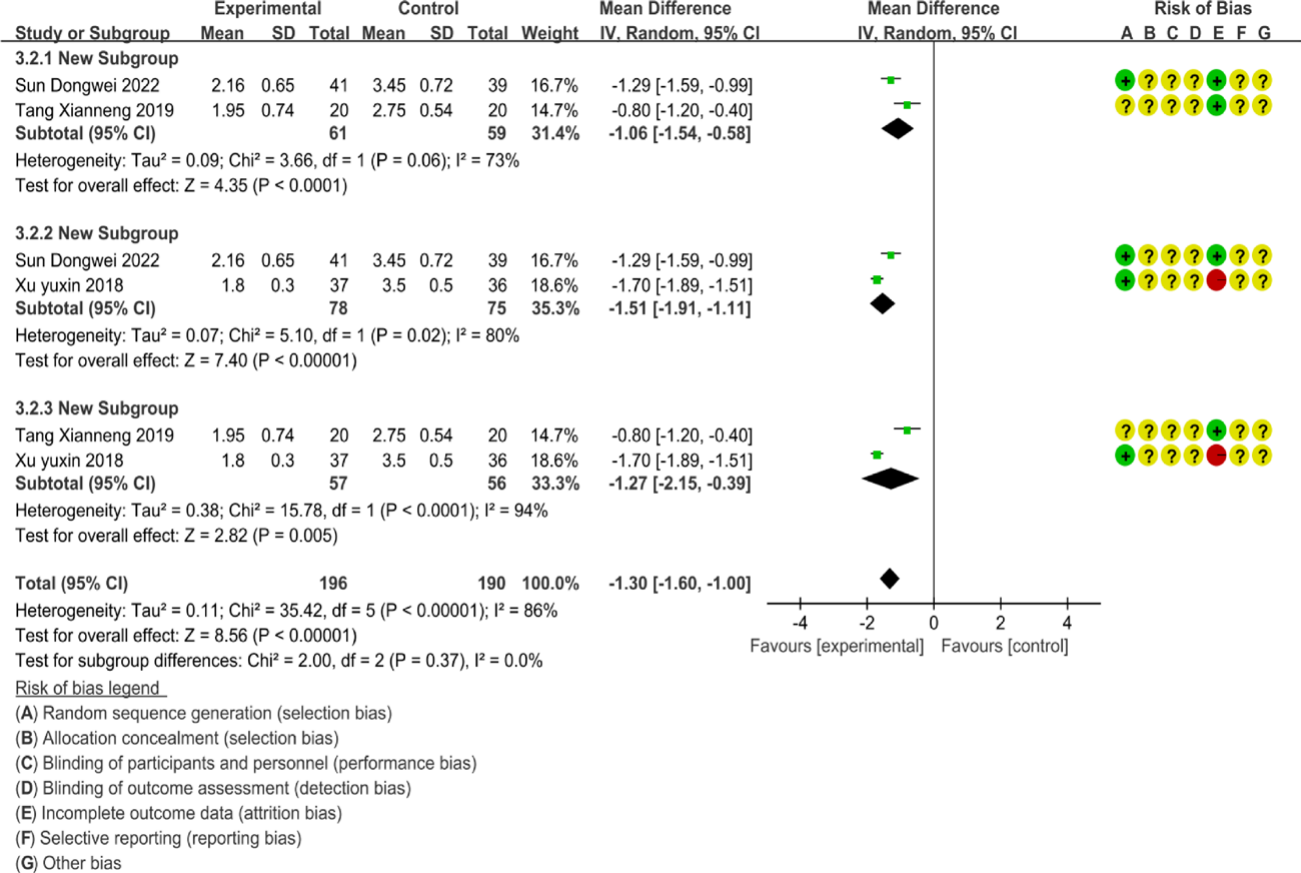
Data graph of FS-14.

The FS-14 fatigue scale is divided into two parts: psychological fatigue evaluation and physical fatigue evaluation. We will analyze the data of the FS-14 fatigue scale from three dimensions: overall score, psychological fatigue, and physical fatigue, As shown in Figures 5–7.

##### 3.4.2.1. FS-14 overall score

Six studies reported FS-14 scores involving 360 subjects.^[21,23,41,47–49]^ The analysis results are shown in Figure 5; The heterogeneity included in the study was high (*P* < .00001, *I*^2^ = 75%), and statistical data was merged using a random effects model; The FS-14 fatigue scale score of massage therapy for chronic fatigue syndrome was significantly lower than other therapies (MD = −1.59, 95% CI = −1.84, −1.34), and the difference was statistically significant (*P* < .00001). Due to the high heterogeneity, we conducted sensitivity analysis and found that none of the studies had a significant impact on heterogeneity. We believe this is due to significant differences in the intervention methods used in the included studies.

##### 3.4.2.2. Mental fatigue score

There are three studies reporting mental fatigue scores, involving 193 subjects.^[23,26,48]^ The analysis results are shown in Figure 6; The heterogeneity included in the study was high (*P* = .07, *I*^2^ = 52%), and statistical data were merged using a random effects model; The psychological fatigue score of massage therapy for chronic fatigue syndrome was significantly lower than other therapies (MD = −0.85, 95% CI = −0.99, −0.72), and the difference was statistically significant (*P* < .00001). Due to the high heterogeneity, we conducted a sensitivity analysis and found that when Xu Yuxin’s study was excluded, heterogeneity was significantly reduced (*P* = .97, *I*^2^ = 0%). We believe this is because, in Xu Yuxin’s study, the control group intervention method was psychological counseling, which had a significant impact on psychological fatigue scores.

##### 3.4.2.3. Physical fatigue score

There are three studies reporting physical fatigue scores, involving 193 subjects.^[23,26,48]^ The analysis results are shown in Figure 7; The heterogeneity included in the study was high (*P* < .00001, *I*^2^ = 86%), and statistical data were merged using a random effects model; The physical fatigue score of massage therapy for chronic fatigue syndrome was significantly lower than other therapies (MD = −1.30, 95% CI = −1.60, −1.00), and the difference was statistically significant (*P* < .00001). Due to the high heterogeneity, we conducted sensitivity analysis and found that none of the studies had a significant impact on heterogeneity. Although it is currently difficult for us to identify the sources of heterogeneity through statistical methods, by exploring the original literature, we can find different sources of heterogeneity. It was found that Tang Xianneng’s research mainly used the technique of regulating tendons and restoring muscles, which is significantly different from the meridian and acupoint massage methods used in the other two studies; In Sun Dongwei’s study, there was a significant difference in the severity of the condition between the control group and the treatment group, while the other two studies did not report the severity of the condition in the subjects. We believe this has also had a certain impact on the score;

#### 3.4.3. Adverse event

1 study^[19]^ reported adverse reactions, including local skin swelling, skin bruising, and nausea in the observation or control groups. The control group in this study received acupuncture treatment, while the observation group received massage treatment. Local skin swelling occurred in 3 cases in the control group and 2 cases in the observation group, Skin bruising occurred in 1 case in the control group and 3 cases in the observation group, Nausea occurred in 2 cases in the control group, while no nausea symptoms were observed in the observation group. These adverse reactions are temporary and do not require medical treatment. No serious adverse reactions occurred; The study did not specify the reasons for these adverse reactions. The remaining 31 studies did not mention adverse reaction events.

### 3.5. Publication bias

**Figure 8.**
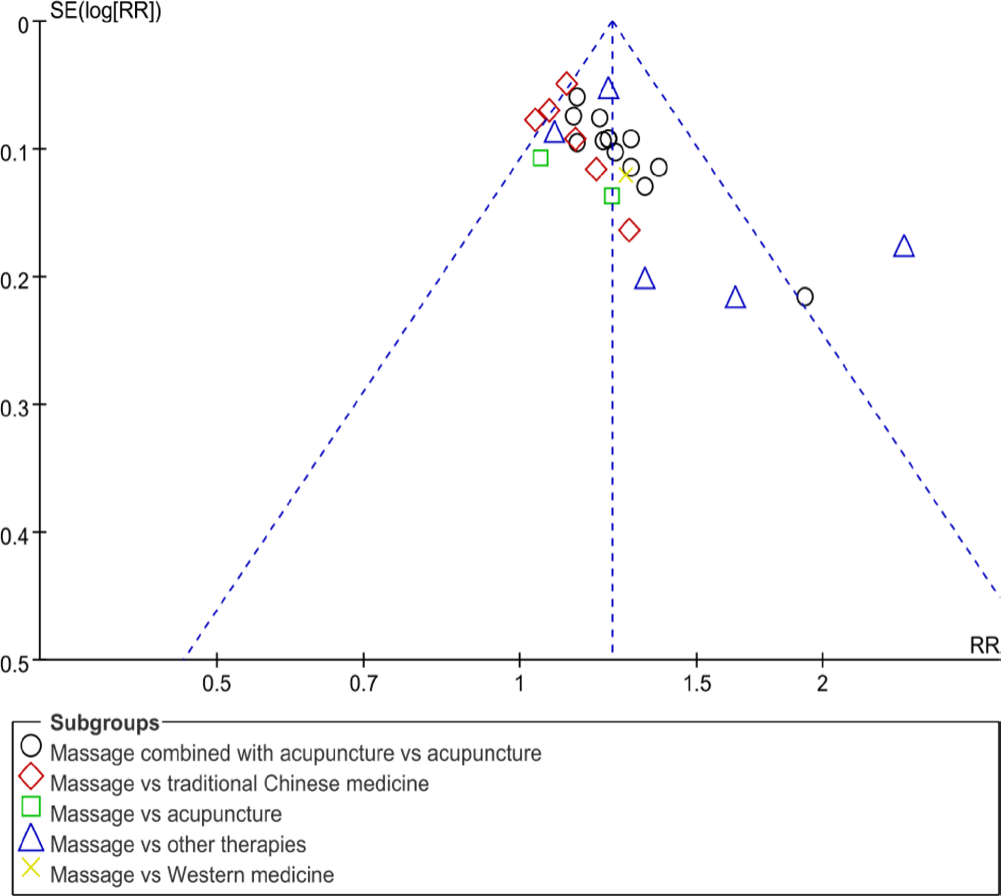
Publication bias.

In this study, 28 studies reported the efficiency analysis. In the publication bias graph, most of these studies were distributed near the top midline and visually symmetrical, indicating no significant publication bias, as shown in Figure 8.

## 4. Discussion

CFS patients often feel distressed due to fatigue, joint pain, low sleep quality, and low mood.^[51]^ At present, Western medicine is unable to explain the cause of this disease. However, from the perspective of traditional Chinese medicine, we believe that there are three causes of CFS: Traditional Chinese medicine believes that emotions have a significant impact on the liver, and patients have poor emotions due to various reasons, leading to liver injury, resulting in common manifestations of CFS such as mental depression, chest and rib pain, breathing difficulties, breast and abdominal distension, abnormal bowel movements, and menstrual irregularities; The patient’s careless diet leads to spleen and stomach injury, weakened digestive function, insufficient nutrition supply, and clinical manifestations such as indigestion, bloating, constipation, and physical fatigue; and Traditional Chinese medicine believes that attention, sleep, and other aspects of the human body belong to the management of the heart. Patients often think excessively and damage the heart, resulting in symptoms such as insomnia, excessive dreaming, forgetfulness, lack of concentration, and overreaction to external stimuli.^[52]^

CFS is mainly caused by the decline of the human immune system, insufficient nutrition supply, and difficulty in recovery over time. Therefore, the treatment approach is to tonify the deficiency and damage to the organs, improve immunity, and increase the nutrition supply. If the patient’s liver is damaged, we should use methods to regulate emotions and select acupoints such as Ganshu, Qimen, and Taichong for massage treatment; If the patient is due to spleen and stomach weakness, we should use acupoints such as Zhongwan, Pishu, and Zusanli for massage treatment; If it is caused by heart injury, we should use acupoints such as Shenmen, Xinyu, and Neiguan for treatment. In addition to selecting corresponding acupoints for massage treatment, the underlying causes should also be removed, such as providing psychological counseling to help patients alleviate depression or anxiety; Educating patients to exercise and enhance their immunity; Customizing recipes for patients, supplementing nutrition, and enhancing treatment effectiveness.

This study merged the effectiveness results and found that regardless of the criteria used to determine effectiveness, the effectiveness rate of the massage treatment group was higher than that of the control group; At the same time, subgroup analysis showed that regardless of the intervention measures used in the control group, the massage treatment group showed significant advantages, indicating that the massage treatment for CFS is effective and superior to other therapies. Tuina therapy provides a comfortable and effective treatment plan for the clinical treatment of CFS. Judging the TCM syndrome type of patients based on their symptoms and signs, selecting massage acupoints is a characteristic of TCM massage treatment, and it also brings great advantages to TCM massage - applying corresponding treatment methods according to the patient’s condition, allowing patients to receive more targeted treatment, and improving the therapeutic effect. According to the results presented in this study, it has been confirmed that tuina therapy is effective in treating this disease. And massage therapy can provide patients with a comfortable and painless treatment plan. At the same time, it can avoid adverse reactions caused by medication treatment and is a relatively safe therapy.

At present, the FS-14 fatigue scale has become the most important indicator for evaluating the condition and efficacy of CFS. So when studying the effect of tuina on clinical symptoms of CFS, we used the FS-14 fatigue scale. This scale was developed by Trudie Chalder from the Psychiatric Research Laboratory of King’s College Hospital in the UK and G. from Queen Mary’s University Hospital Berelowitz and many other experts jointly developed a scale in 1992 for measuring the severity of fatigue symptoms, evaluating clinical efficacy, and screening fatigue cases in epidemiological studies. It is divided into two parts: mental fatigue and physical fatigue, with a total of 14 items.^[17]^ The higher the score, the more severe the fatigue symptoms of the patient. We analyzed the results of the FS-14 fatigue scale and found that the massage treatment group showed lower scores in the overall FS-14 score, mental fatigue score, and physical fatigue score. This indicates that massage therapy can effectively alleviate fatigue symptoms and psychological and emotional problems, with significant advantages in both mental and physical fatigue. Tuina can regulate the psychological and physical symptoms of patients through manual operation, making up for the lack of high acceptance and execution requirements of cognitive behavioral therapy on subjects. At the same time, it reduces the adverse reactions caused by medication. It has high clinical application and promotion value. However, due to the limited number of studies reporting FS-14, mental fatigue score, and physical fatigue score, and the varying design and low homogeneity of these studies, the credibility of the results of this study still needs to be studied.

Tuina therapy, as a noninvasive external treatment, has the advantages of comfort, noninvasive treatment, and minimal adverse reactions. For this purpose, we conducted a statistical analysis of the incidence of adverse reactions. Although only one study reported adverse reactions,^[19]^ and there was no significant difference in the incidence of adverse reactions between the two groups, it is well known that massage therapy can avoid adverse reactions and liver and kidney damage caused by long-term medication, and can avoid more anxiety caused by fear of acupuncture and moxibustion. Tuina therapy can provide a comfortable and safe solution for the clinical treatment of CFS. However, due to insufficient data, more clinical studies are needed in the future to verify the safety of massage therapy.

In the studies we included, a few studies focused on the issue of insomnia in CFS patients and conducted statistics on the Pittsburgh Sleep Quality Index. However, due to the limited number of studies reporting the results of this study and the small number of participants included, we believe that the evidence is not credible and therefore did not conduct an analysis. However through the literature on massage therapy for insomnia, it is not difficult to find that massage therapy also has a definite therapeutic effect on improving sleep quality. In the future, when conducting related research in this field, attention can be paid to the sleep quality of patients, in order to obtain more clinical data and carry out the next step of evidence-based medicine research. At the same time, we believe that Tuina also has a good therapeutic effect on joint pain, but the relevant results have not been reported in the studies we included, indicating that this symptom should also be taken seriously in clinical research in the future.

By carefully reading the literature we have included, we have found that the clinical research designs conducted in this field are not rigorous enough. Most studies have not used scientific allocation methods or blinding methods, although the characteristics of massage therapy make it difficult to achieve double-blind methods. At the same time, there is a lack of uniformity in baseline comparability, data recording, and statistical methods, resulting in high heterogeneity in multiple results. Many symptoms that cause pain in CFS patients have not received sufficient attention and reporting. The results of the report are mainly based on clinical symptoms and subjective scales, with less use of objective indicators. The quality of literature used to report results after the study is also relatively low. This has had a significant impact on evidence-based medicine research in this field and the subsequent standardization of clinical applications. This study is a preliminary evidence-based medicine study in the field of massage therapy for CFS. It not only provides some evidence for the efficacy and safety of massage therapy for CFS but also identifies some urgent issues that need to be addressed in this field of research. Looking into the future, we hope that clinical research conducted in this field can improve the rigor and scientificity of experimental design, pay more attention to other understudied symptoms and signs of CFS, and provide more comprehensive and credible evidence for evidence-based medicine research in the future. At the same time, the modern medical mechanism of this disease is not yet clear, and there are no more accurate objective indicators to diagnose this disease and evaluate its therapeutic effect. Therefore, basic research in this field should also be paid attention to, providing a more scientific basis for future clinical treatment.

## 5. Limitations

The review of this system has some limitations. Most of these studies did not use blinding, which may introduce expected bias. Meanwhile, due to differences in the practitioner’s technique, intensity, and depth, the repeatability of massage is poor, which can cause bias in the experimental results. The studies we included are all Chinese literature, which may be due to the lack of evidence-based medicine validation in the treatment of CFS with massage therapy, which has not yet been widely promoted and applied internationally. There is a certain degree of heterogeneity in the sample size, course of treatment, and combination therapy measures included in different studies. Finally, due to only searching for publicly published studies, the identification of relevant studies may be incomplete.

## Acknowledgments

The authors would like to thank all the researchers in our working group. This work has received support from the Fund in China. Their support is gratefully acknowledged.

## Author contributions

**Conceptualization:** Jingnan Li, qiaoqiao Zeng, Huixin Yan, Yunpeng Bi

**Formal analysis:** Jingnan Li, qiaoqiao Zeng

**Funding acquisition:** Bailin Song

**Investigation:** Yunpeng Bi

**Methodology:** Jingnan Li, Feng piao, qiaoqiao Zeng, Huixin Yan, Yunpeng Bi, Shaobo Zhang

**Resources:** Jingnan Li

**Software:** Feng piao, Shaobo Zhang

**Supervision:** Huixin Yan

**Writing – original draft:** Jingnan Li, qiaoqiao Zeng

**Writing – review & editing:** Bailin Song

## References

[1] Luo L Zhang Y Huang T. A description of the current status of chronic fatigue syndrome and associated factors among university students in Wuhan, China. Front Psychiatry. 2023;13:1047014. 36713904 10.3389/fpsyt.2022.1047014 PMC9877457

[2] Lim EJ Ahn YC Jang ES. Systematic review and meta-analysis of the prevalence of chronic fatigue syndrome/myalgic encephalomyelitis (CFS/ME). J Transl Med. 2020;18:100. 32093722 10.1186/s12967-020-02269-0 PMC7038594

[3] Wu Q Gao J Bai D. Prevalence of chronic fatigue syndrome in China: a meta-analysis. Youjiang Med J. 2020;48:727–35.

[4] Larun L Brurberg KG Odgaard-Jensen J. Exercise therapy for chronic fatigue syndrome. Cochrane Database Syst Rev. 2019;10:CD003200. 31577366 10.1002/14651858.CD003200.pub8 PMC6953363

[5] Afari N Buchwald D. Chronic fatigue syndrome: a review. Am J Psychiatry. 2003;160:221–36. 12562565 10.1176/appi.ajp.160.2.221

[6] Cortes Rivera M Mastronardi C Silva-Aldana CT. Myalgic encephalomyelitis/chronic fatigue syndrome: a comprehensive review. Diagnostics (Basel). 2019;9:91. 31394725 10.3390/diagnostics9030091 PMC6787585

[7] Reid S Chalder T Cleare A. Chronic fatigue syndrome. BMJ Clin Evid. 2011;2011:1101. PMC3275316 21615974

[8] White PD Goldsmith KA Johnson AL.; PACE trial management group. Comparison of adaptive pacing therapy, cognitive behaviour therapy, graded exercise therapy, and specialist medical care for chronic fatigue syndrome (PACE): a randomised trial. Lancet. 2011;377:823–36. 21334061 10.1016/S0140-6736(11)60096-2 PMC3065633

[9] Larun L Brurberg KG Odgaard-Jensen J. Exercise therapy for chronic fatigue syndrome. Cochrane Database Syst Rev. 2016;2:CD003200. 26852189 10.1002/14651858.CD003200.pub4

[10] Jin Y Huang H Zhao N. The application effect of cognitive-behavioral therapy in the rehabilitation of chronic fatigue syndrome. Zhejiang Med J. 2017;39:1036–8 + 1041.

[11] Liu Q Tan J Ling J. Correlation study between TCM constitution and syndrome type in patients with chronic fatigue syndrome. Hubei J Tradit Chin Med. 2013;35:22–3.

[12] Fan B. Massage Therapy. Beijing: China Traditional Chinese Medicine Press, 2016:7–10.

[13] Zhang J. Behavioral intervention guidance and clinical research in chronic fatigue syndrome. Liaoning J Tradit Chin Med. 2009;36:1338–40.

[14] Zhang H Zhu J. Study on the treatment of chronic fatigue syndrome based on the lifting characteristics of viscera qi machine. Shanghai Pharma. 2023;44:15–8.

[15] Available at: https://www.crd.york.ac.uk/prospero/display_record.php?ID=CRD42023481608.

[16] Fukuda K Straus SE Hickie I. The chronic fatigue syndrome: a comprehensive approach to its definition and study. International Chronic Fatigue Syndrome Study Group. Ann Intern Med. 1994;121:953–9. 7978722 10.7326/0003-4819-121-12-199412150-00009

[17] Chalder T Berelowitz G Pawlikowska T. Development of a fatigue scale. J Psychosom Res. 1993;37:147–53. 8463991 10.1016/0022-3999(93)90081-p

[18] Higgins JP Altman DG Gøtzsche PC.; Cochrane Bias Methods Group. The Cochrane Collaboration’s tool for assessing risk of bias in randomised trials. BMJ. 2011;343:d5928. 22008217 10.1136/bmj.d5928 PMC3196245

[19] Shen D Ye Y Xie W. Clinical effect analysis of acupuncture and moxibustion and massage on fatigue syndrome. Smart Health. 2023;9:173–6 + 182.

[20] Xie F Cai Y Tang Y. Clinical study on traditional Chinese medicine treatment of depression based on back meridian massage technique. J Pract Tradit Chin Med. 2023;37:20–3.

[21] Feng W. Observation on the therapeutic effect of abdominal massage on chronic fatigue syndrome of heart and spleen deficiency type. China Urban Rural Enterp Health. 2022;37:166–8.

[22] Han Z Zhao C Zhong X. Analysis of the therapeutic effect of meridian massage combined with balanced cupping on chronic fatigue syndrome of liver depression and spleen deficiency type. Kang Yi. 2022:229–31.

[23] Sun D Wu M Ni X. The clinical efficacy of acupuncture at the back shu point of the five organs combined with massage at the back foot sun bladder meridian in the treatment of chronic fatigue syndrome of liver depression and spleen deficiency type, and its impact on T lymphocyte subsets and blood lipid indicators in patients. Hebei Tradit Chin Med. 2022;44:275–9.

[24] Ren X Li H Lv P. Study on the effect of acupuncture and moxibustion and massage on fatigue syndrome. Worlds Latest Med Inf Dig. 2021;21:303–4.

[25] Shang K Fu Y Liu Q. A study on the immune function regulation of back massage based on chronic fatigue syndrome. J Changchun Univ Tradit Chin Med. 2019;35:909–11.

[26] Tang X Liu J Wei G. Observation on the therapeutic effect of Wechsler maneuver combined with moxibustion on patients with chronic fatigue syndrome. Liaoning J Tradit Chin Med. 2019;46:325–7.

[27] Feng Q. Observation on the therapeutic effect of 13 cases of chronic fatigue syndrome treated with meridian massage combined with stilt walking Modern Health Preservation (Second Half Month Edition). 2018:53–4.

[28] Li J Pu T. To explore the treatment of chronic fatigue syndrome with acupuncture and moxibustion and massage. Special Health. 2018:256.

[29] Li X. The application effect of acupuncture and moxibustion and massage on fatigue syndrome and retrospective analysis of case data. Clin Res Tradit Chin Med. 2018;10:131–2.

[30] Wang J Wang L. The therapeutic effect of tuina on chronic fatigue syndrome. Worlds Latest Med Inf Abstr. 2018;18:135.

[31] Zhang J. The therapeutic effect of tuina on the bladder meridian of the back and waist in patients with chronic fatigue syndrome and its effect on the serum IFN of patients- γ、 TNF- α The influence of content. China Health Nutr. 2018;28:101–2.

[32] Wei G Cheng F. Randomized parallel controlled study of acupuncture and moxibustion+massage in the treatment of chronic fatigue syndrome of liver stagnation and spleen deficiency in young people. J Pract Intern Med Tradit Chin Med. 2017;31:76–8.

[33] Yue C. Clinical observation on the effect of acupuncture and moxibustion and massage on fatigue syndrome. Psychologist. 2017;23:63–4.

[34] Jiang H. Clinical effect analysis of acupuncture and moxibustion and massage on fatigue syndrome. Contin Med Educ. 2016;30:157–8.

[35] Faye W. Study on the effect of acupuncture and moxibustion and massage on fatigue syndrome. Psychologist. 2016;22:150–1.

[36] Fan Y Yan P Wu W. Clinical analysis of 86 cases of fatigue syndrome treated with acupuncture and moxibustion and massage. Ningxia Med J. 2014;36:176–7.

[37] Peng L. Clinical observation on the treatment of chronic fatigue syndrome with traditional chinese medicine tuina combined with modified xiaoyao powder formula. Chin Foreign Med Res. 2013:172–3.

[38] Xiang H. To observe the effect of acupuncture and moxibustion and massage on chronic fatigue syndrome. Clin Res Tradit Chin Med. 2013:39.

[39] Tian H. Clinical study on acupuncture combined with chiropractic therapy for the treatment of chronic fatigue syndrome. Chin Foreign Med Res. 2012;10:13–4.

[40] Wang Y. Observation on the therapeutic effect of Shu Jin Tang combined with massage techniques in the treatment of chronic fatigue syndrome. J Liaoning Univ Tradit Chin Med. 2011;13:220–1.

[41] Wu X Wei X Wang Z. Clinical observation on the treatment of 30 cases of chronic fatigue syndrome (spleen kidney yang deficiency type) with Guben Peiyuan technique. J Changchun Univ Tradit Chin Med. 2011;27:91–92154.

[42] Liu C Lei B. The effect of massage on oxygen free radical metabolism in patients with chronic fatigue syndrome. China Acupunct Moxibustion. 2010;30:946–8. 21246855

[43] Liu S Zhao F. Chiropractic therapy for the treatment of 35 cases of chronic fatigue syndrome. Chin Folk Ther. 2009;17:15–6.

[44] Tang Y. Clinical observation on traditional Chinese medicine health tuina and internal administration of xiaoyao powder modified formula in the treatment of chronic fatigue syndrome. J Liaoning Univ Tradit Chin Med. 2009;11:155–6.

[45] Xu Z Wang J Sun Q. The effect of abdominal massage on motilin levels in patients with chronic fatigue syndrome. J Tianjin Univ Tradit Chin Med. 2006;25:212–4.

[46] Wen B; E Construction. Observation on the therapeutic effect of acupuncture and moxibustion and massage on chronic fatigue syndrome. J Tradit Chin Med. 2005;23:349364.

[47] Qi F Wang Z Dai Y. The effect of tuina on the bladder meridians of the back and waist on the serum IL-6 and CHRM1 levels in patients with chronic fatigue syndrome and clinical efficacy observation. J Hubei Univ Tradit Chin Med. 2020;22:63–6.

[48] Xu Y. Treatment of chronic fatigue syndrome by combining back shu point with head acupoint massage. J Zhejiang Univ Tradit Chin Med. 2018;42:491–3.

[49] Liang F Mei R. Clinical Study on Tongdu Tuina Therapy for Chronic Fatigue Syndrome. Worlds Latest Med Inf Dig (Continuous Electronic Journal). 2014:286–286.

[50] Yao F Fang M Zhu G. The effect of acupoint massage on FS-14 and FAI scores in chronic fatigue syndrome. J Nanjing Univ Tradit Chin Med. 2012;28:222–4.

[51] Aerenhouts D Ickmans K Clarys P. Sleep characteristics, exercise capacity and physical activity in patients with chronic fatigue syndrome. Disabil Rehabil. 2015;37:2044–50. 25512240 10.3109/09638288.2014.993093

[52] Yang X Yang Z. Experience in traditional Chinese medicine treatment of chronic fatigue syndrome. Clin Res Tradit Chin Med. 2020;12:80–3.

